# Properties of the Loess Sediments in Ostrava Region (Czech Republic) and Comparison with Some Other Loess Sediments

**DOI:** 10.1155/2013/529431

**Published:** 2013-12-10

**Authors:** Marian Marschalko, Işık Yilmaz, Lucie Fojtova, David Lamich, Martin Bednarik

**Affiliations:** ^1^VŠB-Technical University of Ostrava, Faculty of Mining and Geology, Institute of Geological Engineering, 17 Listopadu 15, 708 33 Ostrava, Czech Republic; ^2^Cumhuriyet University, Faculty of Engineering, Department of Geological Engineering, 58140 Sivas, Turkey; ^3^Comenius University, Faculty of Natural Sciences, Department of Engineering Geology, Mlynská Dolina, 842 15 Bratislava, Slovakia

## Abstract

This study deals with a methodical identification and evaluation of physical-mechanical properties of one genetic type of geological structure. This is represented by an engineering-geological zone of eolian sediments, which is regionally rather abundant. The paper contributes to a need to identify typical soil properties for widespread geological environments in a particular region and thus add to good engineering geologists and geotechnical engineers' awareness in the region. Such information is much required as it permits comparing results of newly conducted engineering-geological investigations and research with the results characteristic for the region in question. It is vital for engineering geologists and geotechnical engineers to be sufficiently informed on the foundation soil properties of widespread geological environments because of professionalism and higher quality of their work results. Comparing other loess sediment studies worldwide it was discovered that the physical properties of the most abundant clays of low to medium plasticity, sandy clays, and sands as foundation soils vary as for the plasticity index, porosity, natural water content, and bulk density to a certain extent but not as significantly as once expected.

## 1. Introduction

Sediments of an eolian origin are much widespread deposits in Europe, the USA, and Asia. The subject of investigation is their geotechnical properties, determination of the extent of divergence of the individual types of loess [1] or loess in particular regions [2], and examination of hydrocompaction in loess [3]. The characteristics of loess sediments were dealt with by Šajgalik [4], for example, who evaluates the geotechnical properties of Danubian lowland loess rocks in the Carpathians in Slovakia, by Feda [5] in the Prague region in Czech, by Tan [6] in Lanzhow Province in China, by Lin and Wang [7] in Shaanxi Province in China, and by Grabowska-Olszewska [8] in the south of Poland. In the regional scale, physical-mechanical properties of other genetic types of soils or other age were also studied, such as chalky boulder clay in Buckinghamshire in England by Denness [9], glacial deposits by Kazi and Knill [10], or marine clays in Lianyungang in China by Liu et al. [11].

**Figure 1:**
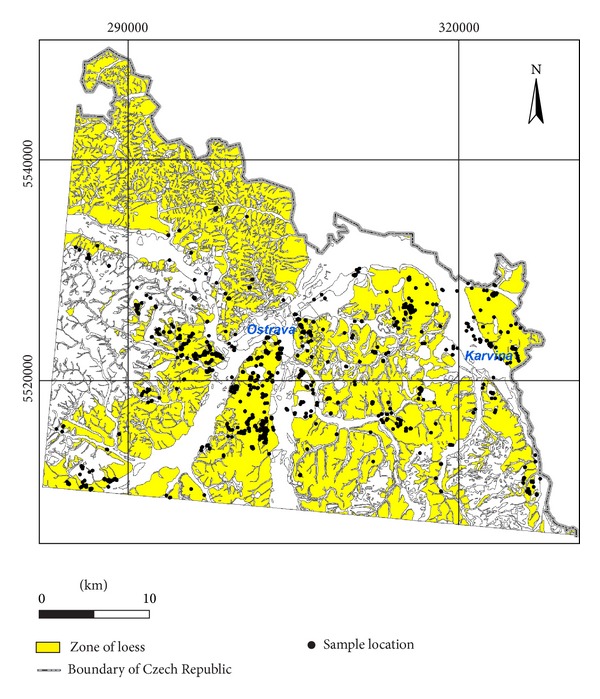
Polygenetic loess sediments in the area of interest.

In this study, geotechnical properties of eolian foundation soils, which covers 46% of study area, are defined and compared with some other loess sediments in different areas. Study area includes Ostrava city, adjacent municipalities, and a part of the Bohumín town (Czech Republic). The compiled database of archive data helped to statistically process laboratory test results determining physical-mechanical properties, which were carried out in 1298 soil samples of an eolian origin (Figure 1). The objective is to evaluate the most widespread sediments in the interest area as foundation soils and to compare their characteristics with available worldwide studies.

This area is characteristic of inhibited mining industry, and thus some percentage is covered with anthropogenic deposits in the form of stock-piles, waste heaps, and so forth. A significant proportion is formed by Quaternary fluvial and eluvial sediments of the Odra and Ostravice Rivers and loess sediments. The underlying Neogene deposits form difficult foundation soils due to a higher percentage of clayey particles which are susceptible to changes in volume.

Unlike other works [12–14] a larger set of foundation soil samples were used for evaluation in the presented study herein. This is the result of the availability of a large archived database of geotechnical and engineering geological investigations where laboratory and in situ tests were conducted on soils in the Czech Republic or other countries. However this database was not considered by means of regional and scientific purposes.

## 2. Stratigraphic and Geological Setting

As stated by Chlupáč et al. [15], the study area is situated in the north of Western Carpathian Foredeep and in contact of the Bohemian Massif and the Carpathian System. The bedrock is made up by a structural floor of Carboniferous age.

From the geomorphological point of view, the study area falls into the Alpine-Himalayan System, subsystem of Carpathians, province of Western Carpathians, and two systems [16]. The subject of the study is Quaternary units which lie on the pre-Quaternary base in the form of Neogene sediments of the Carpathian Foredeep. These sediments were deposited during marine transgression from Eggenburg to Badenian. It is the case of pelitic rocks which have the character of lime clays, greenish-grey to blue-grey colour, brown to brown-grey in higher sections, of stiff consistency all the way to very firm consistency in places of immense solidification. They may have reddish streaks and may contain silty admixture and laminas or interbeds of fine-grained sands. Next, continental sedimentation occurred passing into the Quaternary [15, 17].

In the Pleistocene in the age of Elster and Saale glaciation the area was penetrated by continental glacier from the north and followed by deposition of glacial and poorly sorted gravelly and sandy glaciofluvial sediments and glaciolacustrine clays and sandy clays. In the Upper Pleistocene mainly eolian and deluvial-eolian sediments deposited, the Holocene is represented by fluvial sediments formed by coarse grained soils and small quantity of fine-grained fraction. Holocene is also characterized by overbank sediments such as flood loams and alluvial cones. Deluvial deposits are characterized by loamy sediments and unsorted fragments [18].

The most important sediments covering the majority of the area of interest (Figure 1) are eolian sediments characteristic of uniform sorting and particle size 0.02–0.05 mm [19]. In the Czech Republic eolian sediments are classified into three types: loess containing CaCO_3_, loess loam free of CaCO_3_, and wind-blown sands of particle size 0.1–0.5 mm [15]. In the Ostrava Basin there are predominant loess loam and a low percentage of sandy clays, loam, and sands from the Middle Pleistocene. They are generally yellow-brown, brown to grey-brown and grey or reddish streaks. Their consistencies are mainly firm, and plasticity changes from low to medium.

**Figure 2:**
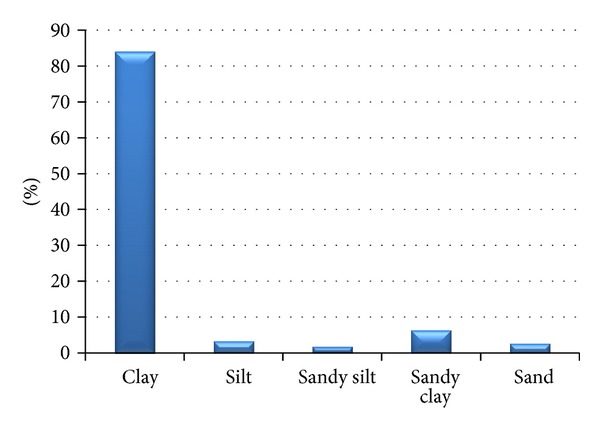
Percentage of loess sediments.

In terms of engineering geology, according to engineering-geological zoning maps [18, 20–22], in the study area there is a zone of polygenetic loess sediments which is the largest and takes up 45.9% (556.1 km^2^) out of the total study area of 1197.4 km^2^. The dominant foundation soil classes are clay (Figure 2). At stiff to firm consistency they have medium bearing capacity and medium compressibility for foundation engineering purposes. It is important to pay attention to collapsibility or susceptibility to collapse.

## 3. Dataset and Methods

The database used in this study includes laboratory test results of 1374 soil samples of an eolian origin which were selected from 10658 soil samples obtained from 6131 boreholes (Figure 1). Effective and total cohesion (*c*_ ef _, *c*_*u*_), internal friction angle (*φ*_ ef _, *φ*_*u*_), oedometric modulus (*E*_ oed _), particle size distribution, bulk density (*ρ*_*s*_), density of solid particles (*ρ*_*n*_), dry density (*ρ*_*d*_), natural water content (*w*), liquid limit (*w*_*l*_), plastic limit (*w*_*P*_), plasticity index (*I*_*p*_), consistency index (*I*_*c*_), porosity (*n*), and degree of saturation (*S*_*r*_) values of the soils were then determined by laboratory tests.

**Figure 3:**
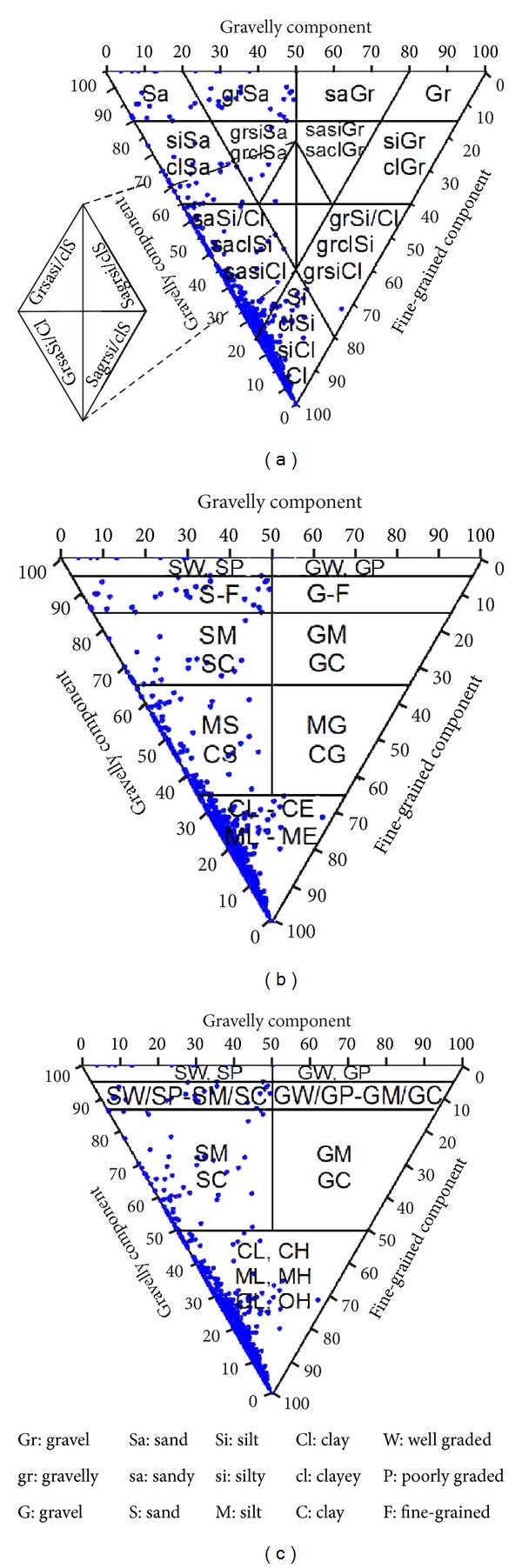
European, Czech, and USCS classification system of soils with marked studied samples: (a) the European soil classification system (ČSN EN ISO 14688-2 Standard); (b) the Czech soil classification system (ČSN 73 1001 Standard); (c) the Unified Soil Classification System (ASTM D2487).

The soils were first classified according to Foundation Soil Classification System (ČSN 73 1001). However this system is similar to Unified Soil Classification System (USCS) (ASTM D2487), the main differences between these classifications systems can be seen in Figure 3.

**Figure 4:**
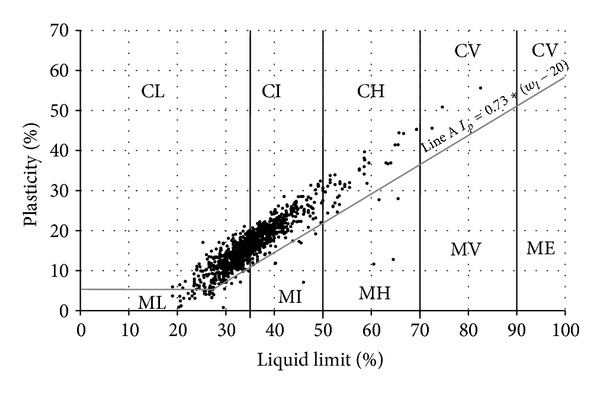
Diagram of plasticity of foundation soil samples.

However the borderline of clay and silt in the European standard is identified by content of clay particles and a line [*I*_*p*_ = 0.73(*w*_*l*_ − 20)] separates silts from clays in USCS (Figure 4). However, in ČSN Standard plasticities of clays and loams are classified into 5 classes (Table 1) according to liquid limit, plasticity of the soils is defined as “low” and “high” by a line which crosses along 50% liquid limit value. In European standard plasticity is classified into 4 classes such as “nonplastic,” “low,” “medium,” and “high,” but their values are not defined.

**Figure 5:**
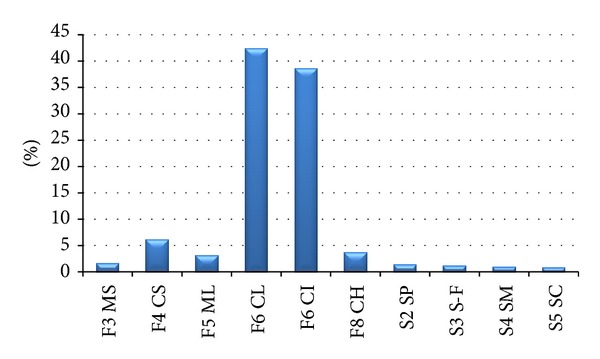
Number of studied samples in the individual foundation soil classes.

1298 soil samples were classified by means of foundation soil classes based on grain size distribution obtained from grain size distribution curves and Atterberg limits (*w*_*l*_, *w*_*p*_, *I*_*p*_). Clays having “low” and “medium” plasticity of class F6 (CL, CI) were found to be the most abundant with 81% - loess loams, 6% are made up by clayey sands of class F4 (CS) and other classes are negligible, that is, below 5% (Figure 5). In order to evaluate the foundation soil class properties statistically, the sandy soil classes were evaluated as one group representing wind-blown sands.

The statistical evaluation was implemented in Minitab application, where verification of normal data set distribution was carried out applying Anderson-Darling test normality combined with histograms and exclusion of outlier values by means of values outside the lower outlier limits [DVH = *Q*_1_ − 1,5(*Q*_3_ − *Q*_1_)] and upper outlier limits [HVH = *Q*_1_ + 1,5(*Q*_3_ − *Q*_1_)] of the set (Pavlík [24]), where *Q*_1_ is the first quartile and *Q*_3_ is the quartile [17]. Intervals of values and average values of physical mechanical properties were evaluated by means of their descriptive statistics.

## 4. Results

**Figure 6:**
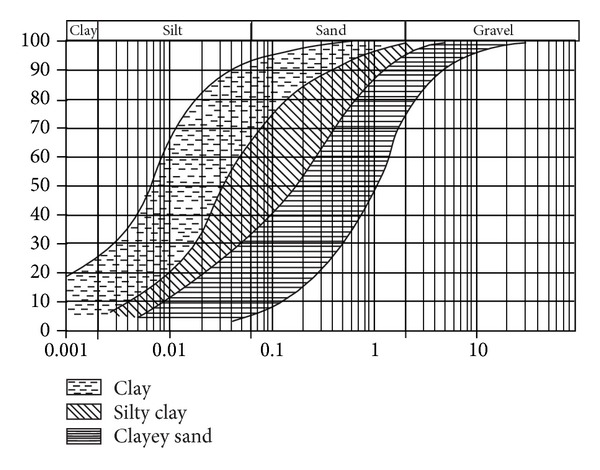
Particles size distributions of studied soil samples.

Loess foundation soils represent less suitable foundation soils than coarse-grained soils. It holds generally true that the bearing capacity decreases, while the compressibility increases due to a higher content (>35%) of clayey and silty particles. Moreover, their behaviour is influenced by the natural water content, which may bring about undesirable phenomena, such as higher frost susceptibility, shrinkage (desiccation), swelling potential, slacking, or collapsibility [25], which is mainly typical for soils of an eolian origin formed by predominant clays of low to very high plasticity. According to ČSN 73 1001 Standard, which reads that the following conditions must be met concurrently, the silty component content is over 60%, the clayey component content is over 15%, *S*_*r*_ < 0.7, and *w*_*l*_ < 32%, and collapsibility in clays was not identified in the study area. Soils are neither susceptible to collapse, which would be indicated by *n* > 40% and *w* < 13% according to ČSN 73 1001 Standard. According to dry density [26] foundation soils of class F8 (CV) may be collapsible as in 15 samples the dry density is lower than 1.28 g·cm^−3^. Particle size distributions of studied soil samples can be seen in Figure 6.

**Figure 7:**
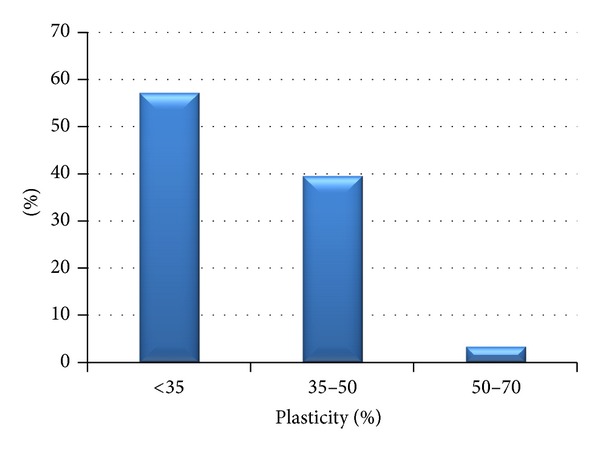
Plasticity according to liquid limit of studied sample (low plasticity *w*_*l*_ < 35%, intermediate *w*_*l*_ = 35–90%, and high *w*_*l*_ = 50–70%).

Figure 7 implies that according to liquid limit, 57.2% of the studied fine-grained samples are low plastic soils (*w*_*l*_ < 35%), 39.5% are medium plastic (*w*_*l*_ = 35–90%), and 3.3% are highly plastic (*w*_*l*_ = 50–70%). Very high plasticity and extreme plasticity were not determined in clays at all. Extremely high plasticity is rare (0.2%). The liquid limit of F6 class clays (CL, CI) ranges from 23 to 47% and of F4 class (CS) it is from 21 to 44%. The interval of loess sands is 17–40%. Feda [5], Tan [6], Lin and Wang [7], and Grabowska-Olszewska [8] stated the liquid limit for loess in various localities—Shaanxi Province and Lanzhow Province in China, Prague region in Czech, and the south of Poland—ranging from 26 to 39%. Compared with the data reported by authors above, the liquid limit values do not much differ, apart from sands that have wider ranges and higher mean value of 32% than 26–28% reported by Lin and Wang [7]. Maximum values as high as 69% for loess are reported by [27] in the environs of Kolín (Czech Republic).

The plastic limit of F6 class clays (CL, CI) ranges from 13.5 to 24% similar to sandy loess of class F4 (CS) having values from 6 to 28%. Other publications state values 10–20% [5, 6, 8], which is a value interval where there are mean values of the ranges stated above. An analogous interval is reported by Lochmann [27], that is, 15–28.7%. The plastic limit for sands is from 12 to 21%.

The plasticity index range is 4–29% for clays (F6 CL, CI) and 11.5–26% for sandy clays (F4 CS). The mean value is 16%, which corresponds to loess near Prague [5]. Maximum values in the environs of Kolín are higher as they reach 45% (Lochmann, [27]). In China the plasticity index values are slightly lower, namely, 10–14% [6, 7]. Lin and Wang [7] state plasticity index of 8–10% for sandy loess in China, which are lower values than in the studied samples, that is, 3–21% with the mean value of 14%.

Consistency of eolian sediments is predominantly solid (55%), evenly stiff, and very firm (20%) and in rare cases the consistency is soft and very soft.

The water content in loess sediments of class F6 (CL, CI) ranges from 13 to 30% with the mean value of 20% and for class F4 (CS) it is 10–29% with the mean value of 17%. In general, the values correspond to the water content in loess in other localities, where only Tan [6] reports lower values, that is, 10–11%. Loess sands have 6–24% water content with the mean value of 18.%, which is considerably higher value than that reported by Lin and Wang [7], that is, 9–13%.

Specific gravity is consistent for fine-grained foundation soils F6 (CL, CI) and F4 (CS), that is, 2.65–2.75 g/cm^3^, which are identical values to those reported by Bell [19], Lochmann [27], and Grabowska-Olszewska [8]. Sands have similar specific density as clayey loess, that is, 2.60–2.73 g/cm^3^.

The interval of bulk density, 1.8–2.3 g/cm^3^, is identical to classes F4 (CS) and F6 (CL, CI) in the area of interest also to loess from the environs of Kolín (Lochmann [27]), but it is higher compared with Polish loess sediments of 1.54–2.12 g/cm^3^ [8], the loess interval in China of 1.45–1.85 g/cm^3^ [7], or the values of Peorian loess, 1.34–1.55 g/cm^3^, according to Lutenegger and Hallberg [28]. The specific gravity for sands, 1.86–2.09 g/cm^3^, is lower than for sandy loess sediments in China, 1.59–1.68 g/cm^3^ [7].

In the studied samples, the values of dry density were measured identical to class F6 (CL, CI) and F4 (CS) in the interval of 1.44–1.9 g/cm^3^, which agrees to loess in other localities (Lochmann [27]) [6, 8]. Sands have dry density from 1.52 to 1.74 g/cm^3^.

Very similar values of porosity in the interval of 30–49% are manifested by fine-grained loess sediments in the study area and in the European regions. In the Chinese provinces the porosity is higher, that is, 43–53% [6, 7], which corresponds to lower bulk density. It is analogous with sandy foundation soils, whose porosity is 32–41% in the area of interest and 44–48% in Shaanxi Province [7].

The mechanical properties [7, 19] were determined in clays of low to medium plasticity (F6 CL, CI). Oedometric modulus was measured in 134 studied samples, effective shear strength parameters in 87 samples, and total shear strength parameters in 35 samples. They are important parameters to determine the geotechnical behaviour [29] of the geological environment interacting with engineering-geological structures or in connection with anthropogenic changes. Considering the fact that mechanical properties are influenced by consistency, it is advisable to classify the studied samples according to consistency and later evaluate their parameters. As the number of firm consistency studied samples is much lower than the samples of stiff consistency, this influence has not been proved and the property values are identical in both the cases. The oedometric modulus is determined as 4.4–18.5 MPa with the mean value of 9.6 MPa for the load factor 100–400 kPa, which corresponds to Lochmann [27]. The effective cohesion and internal friction angle range from 4 to 33 kPa and from 20 to 30°, respectively. The measured total shear strength parameters range from 35 to 137 kPa and from 0 to 30°, respectively.

## 5. Conclusions

Within engineering-geological investigations, 1298 soil samples of an eolian origin were drawn in the study area, having an area of 1211.4 km^2^. For the study, they were analyzed for basic physical-mechanical properties of predominantly fine-grained soils, made up in 81% by clays of low to medium plasticity of class F6 (CL, CI)—loess loams, 6% of clayey sands of class F4 (CS), and rare other classes that is below 5%. The figure of clay proportion must be understood as biased, as the statistical results have been influenced by the documenting geologists' subjective choice of sampling points in the bore profiles. From the point of view of spatial distribution, the high proportion of clays is affected by a limiting factor of purpose and need to situate engineering work and it is not representative for the overall geological environment.

Evaluating the character of physical properties of the studied samples in the area of interest, it may be stated that their values well correspond to other works dealing with the characteristics of loess sediments worldwide, such as in Feda [5] in the Prague region in Czech, Tan [6] in Lanzhow Province in China, Lin and Wang [7] in Shaanxi Province in China, and Grabowska-Olszewska [8] in the south of Poland. Wind-blown sands make an exception as they differ in the liquid limit values, plasticity index, and water content from loess sands in the Shaanxi Province in China [7]. Loess clays of class F6 (CL, CI) and sandy clays (F4 CS) have a higher bulk density and related lower porosity than other compared loess. The properties of the studied sands and clayey foundation soils agree to a certain extent, for example, in case of bulk density, which is confirmed by the same course of deposition and high homogeneity of the environment.

Mechanical properties represented by the oedometric modulus and shear parameters were studied only in class F6 (CL, CI).

The study results imply local characteristics of a regional importance which may be produced for any region applying a similar methodology. On the grounds of the comparison of the foundation soils it may be stated that loess has identical or very similar properties independently of the place of occurrence. The regions that are less distant to one another have more similar properties of the given genetic type. It also confirmed that the higher the number of studied property values is, the more accurate they are, which arises from the comparison with Lochmann [27] who characterizes loess in the environs of Kolín in the Czech Republic by means of laboratory analyses results of maximum of 107 soil samples.

## Figures and Tables

**Table 1:** Classification of the plasticity of soils according to ČSN Standard 2003 [23].

Liquid limit (%)	>90	90–70	70–50	50–35	<35
Plasticity class	Extremely high	Very high	High	Medium	Low
